# Associations between fetal size, sex and placental angiogenesis in the pig^†^

**DOI:** 10.1093/biolre/ioy184

**Published:** 2018-08-18

**Authors:** Claire Stenhouse, Charis O Hogg, Cheryl J Ashworth

**Affiliations:** Developmental Biology Division, The Roslin Institute and Royal (Dick) School of Veterinary Studies, University of Edinburgh, Midlothian, UK

**Keywords:** fetal growth, intrauterine growth restriction (IUGR), porcine, pregnancy, reproduction, sexual dimorphism, vascularity

## Abstract

Inadequate fetal growth cannot be remedied postnatally, leading to severe consequences for neonatal and adult development. It is hypothesized that growth restriction occurs due to inadequate placental vascularization. This study investigated the relationship between porcine fetal size, sex, and placental angiogenesis at multiple gestational days (GD). Placental samples supplying the lightest and closest to mean litter weight (CTMLW), male and female Large White X Landrace fetuses were obtained at GD30, 45, 60, and 90. Immunohistochemistry revealed increased chorioallantoic membrane CD31 staining in placentas supplying the lightest compared to those supplying the CTMLW fetuses at GD60. At GD90, placentas supplying the lightest fetuses had decreased CD31 staining in the chorioallantoic membrane compared to those supplying the CTMLW fetuses. The mRNA expression of six candidate genes with central roles at the feto-maternal interface increased with advancing gestation. At GD60, *ACP5* expression was increased in placentas supplying the lightest compared to the CTMLW fetuses. At GD45, *CD31* expression was decreased in placentas supplying the lightest compared to the CTMLW fetuses. In contrast, *CD31* expression was increased in placentas supplying the lightest compared the CTMLW fetuses at GD60. In vitro endothelial cell branching assays demonstrated that placentas supplying the lightest and male fetuses impaired endothelial cell branching compared to placentas from the CTMLW (GD45 and 60) and female fetuses (GD60), respectively. This study has highlighted that placentas supplying the lightest and male fetuses have impaired angiogenesis. Importantly, the relationship between fetal size, sex, and placental vascularity is dynamic and dependent upon the GD investigated.

## Teaser

Placentas supplying the lightest and male porcine fetuses have impaired angiogenesis compared to their normally grown and female littermates. The relationship between fetal size, sex, and placental vascularity is dependent upon the gestational day investigated.

## Introduction

Due to the superficial attachment of the porcine placenta to the maternal endometrium [1], extensive angiogenesis must occur at the feto–maternal interface to allow sufficient nutrient transfer between the mother and the fetus. Around the time of conceptus attachment (gestational day (GD) 13–18), an initial wave of uterine angiogenesis occurs [2], followed by a second phase of angiogenesis in mid-gestation to increase the availability of nutrients to the exponentially growing fetus [3].

It has been shown in a number of species that uterine and umbilical blood flow both increase as gestation progresses [4–9], which is an important mechanism to increase nutrient transfer to the fetus [4, 10]. In the pig, the available attachment area and the density of the vascular supply available for each individual feto-placental unit must increase to meet the increasing requirements of the growing fetuses [11].

Placental efficiency is usually defined as the weight of the fetus/piglet per mass of placenta [12, 13]. Efficient placentas have been shown to be shorter with a denser vascular supply when compared with less efficient placentas [14]. It has been demonstrated that highly prolific pig breeds, such as Chinese Meishan, have smaller placentas with an increased vascular density contributing to increased placental efficiency compared to less prolific commercial European breeds [12, 15]. Further, it has been shown that smaller, more efficient placentas have increased vascularity and expression of vascular endothelial growth factor (VEGF) compared to less efficient placentas [3].

Large within-litter variation in placental efficiency has been observed and is proposed as a significant contributor to the large within litter variation in birthweight observed in pigs [12, 16–18]. In addition to the large variation in fetal and birth weight observed, pigs are the most extreme example of naturally occurring intrauterine growth restriction (IUGR) among the mammalian species studied to date [19–21]. It has been suggested that IUGR may occur due to decreased maternal–fetal blood flow and/or impaired angiogenesis at the feto–maternal interface [8, 17, 22]. Appropriate regulation of placental angiogenesis is therefore essential for the establishment and maintenance of successful pregnancy in pigs.

Recent studies have revealed sexual dimorphism in human placentas [23, 24], with fetal sex influencing the expression of placental genes and the inflammatory response [25, 26]. Additionally, on occasions where human pregnancy is complicated by preeclampsia or IUGR, male offspring have increased perinatal mortality and morbidity [26, 27]. In pigs, it is proposed that male new-born piglets have a survival disadvantage compared to their female littermates [28]. However, despite the intriguing data to suggest sexual dimorphism in placental development in humans, this area remains poorly understood in the pig.

In this study, the mRNA expression of six candidate genes with proposed roles in regulating vascularity and remodeling of the feto–maternal interface was investigated. Uteroferrin (*ACP5*) is a glycoprotein which is secreted by the glandular epithelium (GE) of the porcine endometrium in response to progesterone (P4) [29]. It transfers iron to the developing fetus [29–33] and acts as a hematopoietic growth factor [34]. Platelet and endothelial cell adhesion molecule 1 (*CD31*) is an endothelial cell marker expressed at the junctions between endothelial cells [35, 36] and has been suggested to be involved in angiogenesis by regulating endothelial cell migration [37–42]. It has been demonstrated in both an experimental rat model [43] and in porcine placentas in late gestation [44] that IUGR fetuses have increased hypoxia inducible factor 1 alpha subunit (HIF1A) expression compared to those supplying their normally grown littermates. The endoglycosidase heparanase (HPSE) degrades heparan sulfate [45] to allow remodeling of the extracellular matrix of the placenta to meet the ever-increasing demands of the exponentially growing fetus. HPSE has a proposed role in angiogenesis, inducing endothelial cell migration and proliferation by degrading the basement membrane to allow the invasion and migration of endothelial cells and the release of the angiogenic factors basic fibroblast growth factor (bFGF) and VEGF [46, 47]. Conceptus secreted estradiol upregulates the expression of prostaglandin F2 alpha receptor (PTGFR) in the porcine endometrium in the blood vessels, luminal epithelium, and GE [48, 49]. The action of prostaglandin F2 alpha (PGF2α) binding to the PTGFR on the endometrium results in increased production and expression of VEGFA by the endometrium. Binding of VEGFA to its receptors increases angiogenesis in the endometrium by inducing proliferation and migration of endothelial cells [48]. In the porcine placenta, the fetal vessels expressed VEGF and VEGF receptors and, from GD30 onwards, intense protein expression was observed in trophoblast cells [50]. Further, Charnock-Jones et al. [51] demonstrated that VEGF binds to VEGF receptors in the endothelial cells of the endometrium, and to the small capillaries found in the bilayer, suggesting that VEGF may promote angiogenesis of the small capillaries in the bilayer.

Endothelial cells line the interior of blood vessels and are exposed to circulating blood in the lumen of the vessel. Several of the candidate genes of interest are known to induce endothelial cell proliferation and migration during angiogenesis. Considering the results of the first two experiments described herein, the angiogenic potential of conditioned media generated from GD45 and 60 placental samples, supplying fetuses of different size and sex, was assessed using an in vitro endothelial cell branching assay [52–57].

## Materials and methods

All procedures were performed with approval from The Roslin Institute (University of Edinburgh) Animal Welfare and Ethical Review Board and in accordance with the UK Animals (Scientific Procedures) Act, 1986.

### Experimental animals and sample collection

Large White X Landrace gilts (age 11–14 months; n = 31) were observed daily for signs of estrus and were housed in groups of 6–8 animals per pen. Estrous cyclicity and ovarian function were controlled in accordance with routine normal practice at The Roslin Institute Large Animal Unit. In a subset of gilts (distribution between the GD investigated indicated in Supplementary Table S1), estrus was synchronized by daily feeding of 20 mg Altrenogest (Regumate, Hoechst Roussel Vet Ltd) for 18 days followed by injection of pregnant mare serum gonadotropin (Intervet UK Ltd) and human chorionic gonadotropin (Intervet UK Ltd) [58]. All gilts were inseminated twice daily for the duration of estrus with semen from one of four boars (Large White). The boars used were equally distributed between gilts at the different GD to minimize the effect of boar. The first day of insemination was assigned as GD0. Placental samples were obtained at GD30, 45, 60, and 90 (n = 6, 6, 11, and 8 respectively). Gilts were euthanized with sodium pentobarbitone 20% w/v (Henry Schein Animal Health) at a dose of 0.4 ml/kg by intravenous injection via a cannula inserted in the ear vein.

Following confirmation of death, mid-ventral incision revealed the reproductive tract. The tract was lifted from the body cavity and placed in a dissecting tray. Both uterine horns were dissected, from the ovary towards the cervix. The uterine lumen was occluded between each feto–placental unit by tying with string to ensure that placental tissues associated with particular fetuses could be identified later. All fetuses were identified as “live” or “dead” based on their morphology at the time of dissection and were weighed. At GD45, 60, and 90, fetal sex was determined morphologically. DNA was isolated from the GD30 fetuses using the DNeasy Blood and Tissue DNA extraction kit (Qiagen), and PCR was performed for the sex-determining region Y (Sry) region of the Y chromosome to sex the fetuses as described previously [59]. Primers for the homologous zfx/zfy regions of the sex chromosomes served as a positive control (forward primer 5΄-ATAATCACATGGAGAGCCACAAGCT-3΄; reverse primer 5΄-GCACTTCTTTGGTATCTGAGAAAGT-3΄) [60]. A no-template control and postnatal porcine testicular and ovarian DNA were run as controls. The PCR products were visualized by electrophoresis on a 1.2% (wt/vol) agarose 1 X SybrSafe (ThermoFisher) gel with 1 X Tris/Acetic Acid/EDTA (TAE) buffer. Supplementary Table S1 summarizes the characteristics of the litters used in this study.

The lightest and the closest to the mean litter weight (CTMLW) fetus (GD30) of both sex (GD45, 60, and 90) were identified based on fetal weight. From the anti-mesometrial side, placental samples were taken from each feto-placental unit of interest. Placental samples were snap-frozen in liquid nitrogen and stored at –80°C for RNA extraction or fixed in Bouin's (Sigma Aldrich) for histology.

### Experiment 1: Analysis of placental vascularity by immunohistochemistry for CD31

#### Immunohistochemistry

For this experiment, placental samples from GD45, 60, and 90 (n = 6, 7, and 5 litters, respectively) were used. The samples were fixed with Bouin's overnight at room temperature and changed daily for approximately 1 week in 70% ethanol (Genta Medical). The samples were transferred into labeled tissue processing cassettes (Simport) and processed using a tissue processor (ASP3005, Leica) by passing through graded ethanol (70%, 95%, and 99%; Genta Medical) and xylene (Genta Medical). The samples were embedded in paraffin wax (Fisher Scientific) and 5 μm sections were cut and placed on polysine microscope slides (Fisher Scientific).

Following dewaxing and heat-induced epitope retrieval in 0.01M sodium citrate (Vector Laboratories), endogenous peroxidase activity was blocked by incubating slides with 0.3% H_2_O_2_ (Sigma Aldrich) in methanol. Nonspecific binding sites were blocked by incubation with normal goat serum (Vectastain Elite ABC kit; Vector Laboratories). Sections were incubated with rabbit anti-CD31 antibody (ab28364; Abcam) at a 1:100 dilution (Supplementary Table S2) or with rabbit immunoglobulin G (RIgG) (Vector Laboratories) (equivalent total protein concentration) as a negative control. The slides were incubated in a humidified chamber at 4°C overnight, washed in phosphate buffered saline (PBS), and incubated for 30 min at room temperature with a biotinylated anti-rabbit IgG secondary antibody (Vectastain Elite ABC kit; Vector Laboratories) at a dilution of 1:200 in 1.5% normal goat serum (Supplementary Table S2). Sections were incubated with Vectastain Elite ABC reagent (Vectastain Elite ABC kit; Vector Laboratories) for 30 min, before incubation with the Novared peroxidase substrate (Vector Laboratories) for 5 min. Sections were counterstained with hematoxylin and dehydrated in a graded series of ethanol and xylene (70%, 95%, and 99% ethanol; 99% ethanol 1:1 with xylene, and absolute xylene; Genta Medical). The sections were imaged using the NanoZoomer slide scanner (Hamamatsu).

#### Image analysis

All image analyses were performed using ImageJ. The stromal regions and the chorioallantoic membrane (CAM) region of the placenta were analyzed separately. At GD45, six images were taken of stromal regions which did not contain CAM regions at ×20 magnification from the slide scans of two sections. At GD60, four images of the same regions were taken at ×10 magnification. The number of blood vessels present was quantified for each image and a grid (21,500 μm^2^) was superimposed on the image. The internal and external blood vessel diameters were measured in triplicate for the blood vessels present in every second square. The mean internal diameter was subtracted from the mean external diameter to give the vessel wall thickness. The images were split into red, green, and blue channels, and the percentage staining was quantified using the green channel at a threshold of 136 pixel intensity.

To investigate the percentage staining of the CAM at GD45, 60, and 90, three images at ×10 magnification were taken from two sections. The images were split into red, green, and blue channels and, using the freehand drawing tool on the green channel, the CAM area was selected, and the percentage staining quantified (Threshold 136 pixel intensity).

### Experiment 2: Analysis of candidate gene expression by qPCR

The mRNA expression of candidate genes was investigated by qPCR in placental samples supplying fetuses of different size and sex throughout gestation.

#### Total RNA extraction and cDNA synthesis

RNA was extracted from 20 to 50 μg of snap-frozen placental samples [61], with the addition of a DNase treatment step (RNase-free DNase, Qiagen), as per the manufacturer's instructions. The RNA was quantified, and the quality assessed spectrophotometrically using a Nanodrop ND-1000 (Labtech International Ltd) and electrophoretically using a Tapestation 2200 (Agilent Technologies). The mean A260/A280 and RNA Integrity Number Equivalent (RINe) for samples within each GD are detailed in Supplementary Table S3. Extracted RNA was stored in a freezer at –80°C until required. In the case of a sample having a RINe value below the ranges detailed in Supplementary Table S3, the sample was excluded from the analyses.

Complementary DNA (cDNA) was prepared from 0.3 μg of RNA with SuperScript III reverse transcriptase (Life Technologies) following the manufacturer's instructions. Each reaction contained 250 ng random primers (Promega) and 40 units RNaseIn (Promega). Negative controls without reverse transcriptase were included to check for genomic contamination. Reverse transcription was performed in duplicate for each sample and pooled, and the cDNA was stored at –20°C until required.

#### Relative expression of candidate genes in placental samples

Quantitative PCR was performed on a Stratagene MX3000 instrument using Platinum SYBR Green SuperMixUTG (Life Technologies) using cDNA from placentas at GD30, 45, 60, and 90 (n = 6, 6, 6, and 8 litters, respectively). The placental samples were associated with the lightest and CTMLW fetuses at GD30, and the lightest and CTMLW fetuses of both sex at GD45, 60, and 90. The final concentrations of magnesium, ROX reference dye, and each primer were 3 mM, 50 nM, and 400 nM, respectively in a 25 μl reaction volume. All qPCRs were carried out at an annealing temperature of 60°C and dissociation curves consisting of single peaks were generated. The mRNA expression of six candidate genes of interest was quantified: *ACP5* [61], *CD31, HIF1A* [60], *HPSE* [62], *PTGFR* [49], and *VEGFA* [60]. Two reference genes were used: hypoxanthine phosphoribosyl-transferase 1 (*HPRT1*) and TATA box binding protein 1 (*TBP1*). These reference genes were identified as having stable expression in placental samples by analysis of 11 previously identified reference genes [63, 64] using geNORM V3.5 (Ghent University Hospital, Centre for Medical Genetics). The primer sequences for all genes investigated in experiment 2 are detailed in Supplementary Table S4.

Serial dilutions of pooled cDNA ranging from 1:5 to 1:640 in nuclease-free water were used as standards. Sample cDNA was diluted 1:25 and 5 μl of sample, standard, or control were added per well. Each plate contained duplicate wells of a no-template control, standards, sample cDNA, and reverse transcriptase blanks. Data were analyzed using qbase + software V3.0 (Biogazelle). A target and run specific strategy was employed and the results, normalized to the two reference genes, were scaled to the minimum sample. The mean slope, intercept, PCR efficiency, and R^2^ values are detailed in Supplementary Table S5.

### Experiment 3: Analysis of in vitro endothelial cell branching in response to placental conditioned media

#### Generation of conditioned media and assessment of tissue viability following culture

Considering the results of experiments 1 and 2, which suggested dynamic changes in placental vascularity between GD45 and 60, the angiogenic potential of placental samples at these GD was investigated further. Conditioned media was generated from GD45 and 60 (n = 3 and 6 litters, respectively) placentas supplying fetuses of different size and sex. Within litter, placental samples were taken from the lightest and CTMLW fetus of the same sex. At GD45, the placental samples were all obtained from female fetuses, due to limited availability of male samples. At GD60, half of the samples were from placentas supplying male fetuses and the remainder were from placentas supplying female fetuses.

Following dissection of the uterus, a sample of whole uterus from the feto–placental units of interest were placed in a jar containing sterile PBS supplemented with Penicillin-Streptomycin (PenStrep; Life Technologies) and transported to the laboratory. The placenta was dissected from the underlying endometrium and myometrium. Pieces of tissue were cut using a 17 mm corer and representative pieces of tissue were weighed to ensure tissue cutting was consistent. The tissue was placed in a 6 well plate (NUNC, ThermoFisher) containing 8 ml of media (DMEM-F12; Sigma Aldrich) supplemented with 10% heat-inactivated newborn calf serum (NBCS; Life Technologies) and PenStrep and cultured for 18 h, after which the media was removed and stored at –80°C until required.

The cultured tissue explants were fixed overnight at 4°C in 4% paraformaldehyde, placed in histological cassettes, and dehydrated by passing through graded ethanol and xylene (Genta Medical) before being embedded in paraffin wax. From each specimen, 5 μm sections were cut and placed on polysine microscope slides (Fisher Scientific). To ensure that the cultured samples were still proliferating following culture, the sections were stained for the proliferation marker Ki67. Immunohistochemistry was performed as described in experiment 1, with the primary antibody (rabbit anti-Ki67 ab15580; Abcam) at a 1:200 dilution (Supplementary Table S2) or with RIgG (Vector Laboratories) (equivalent total protein concentration).

#### Immunocytochemistry of G-1410 endothelial cells for CD31 and vWF

The G-1410 immortalized pig endothelial cell line generated by Chrusciel et al. [65] was used for the branching assays. To ensure the G-1410 cells expressed markers of endothelial cells, immunocytochemistry was performed for the endothelial cell markers CD31 and von Willebrand Factor (vWF). The adherent cells of interest were pregrown on poly-D-lysine coated glass coverslips (Neuvitro) in a 12-well plate (Eppendorf). Prewarmed 4% paraformaldehyde was added to the wells and the cells were incubated with this at room temperature for 2 min before washing twice with PBS. The cells were incubated in prewarmed 4% paraformaldehyde at room temperature for a further 15 min, which was followed by three PBS washes. Ice-cold absolute methanol (Fisher Scientific) was added and the cells were incubated at –20°C for 10 min. The methanol was removed, and the cells were permeabilized by incubation with 0.3% Triton-X (Sigma Aldrich) in PBS for 5 min at room temperature. This step was followed by three PBS washes and a 60-min block step at room temperature in protein block solution (DPB-125, Springbio). The cells were incubated overnight at 4°C with the primary antibody diluted in PBS with 5% normal goat serum (CD31 ab28364 (Abcam), 1:20 dilution; vWF ab6994 (Abcam), 1:400 dilution) (Supplementary Table S2). A RIgG negative control (Vector Laboratories) at an equivalent protein concentration to the primary antibody of interest and a secondary antibody only (PBS with 5% normal goat serum) control were set up and incubated overnight at 4°C. Three PBS washes were performed before a 60-min incubation with the fluorescence-labeled secondary antibody diluted in PBS with 5% normal goat serum (Alexa Fluor® 488 goat anti-rabbit (H + L). 1:1000 dilution; Thermo Fisher) (Supplementary Table S2). The coverslips were washed twice in PBS, before counterstaining with 6 ng/ml 4΄,6-diamidino-2-phenylindole (DAPI) solution (Novus Biologicals LLC) for 10 min. The coverslips were rinsed with dH_2_O and mounted with ProLong Gold Antifade reagent (ThermoFisher Scientific) using concave slides (Marienfeld). The cells were imaged using a LSM 710 confocal microscope at ×40 magnification (Zeiss).

#### PCR of cDNA from G-1410 endothelial cells for endothelial cell markers

RNA was extracted from the endothelial cells using the RNeasy Mini-Kit (Qiagen) as per the manufacturer's instructions, and cDNA was synthesized as detailed in experiment 2. PCR was performed using the cDNA for the endothelial cell markers: *CD31*, two isoforms of *VEGFA* (*VEGF120* and *VEGF164*), and vascular endothelial growth factor receptor 1 (*VEGFR1)*. The reference gene Beta-2-microglobulin (*B2M1*) was used as a positive control. The primer sequences for all genes are detailed in Supplementary Table S6. The PCR was performed under the same conditions as the SRY PCR described previously. Electrophoresis of the PCR products was performed using a 2% (w/v) 1 X SybrSafe (ThermoFisher) agarose gel with 1 X TAE buffer.

#### Matrigel branching assay

The bottom of wells of a 24-well tissue culture plate (Eppendorf) were coated with 300 μl Growth Factor Reduced Matrigel Basement Membrane (Corning) and incubated at 37°C and 5% CO_2_ for 30 min to solidify. G-1410 cells cultured in T75 flasks (Eppendorf) at approximately 80% confluency were serum starved (M199 (Sigma Aldrich) supplemented with PenStrep) for approximately 24 hours and dissociated from the flask by incubation with TryPLE (Life Technologies, Paisley, UK). The reaction was neutralized by the addition of media (DMEM-F12 supplemented with 10% NBCS and PenStrep), and the cell suspension was centrifuged for 5 min at 400 g. The cell pellet was suspended in the required volume of media which would allow 50,000 cells to be added to the well in a 10 μl volume.

The conditioned media was thawed in a water bath (37°C) immediately prior to use and prepared by mixing 200 μl of the conditioned media with 100 μl of branching assay media (DMEM-F12 supplemented with 10% NBCS and PenStrep). To this, the 10 μl cell suspension (50,000 cells) was added and the cells were gently mixed by pipetting. The cells were added to the wells of interest, with each sample being assayed in duplicate. In addition to the conditioned media of interest, a media only control (300 μl DMEM-F12 supplemented with 10% NBCS and PenStrep), and a positive control (300 μl DMEM-F12 supplemented with 10% NBCS and PenStrep containing Recombinant Human EG-VEGF (20 ng/ml) (Peprotech) were assayed in duplicate.

The cells were allowed to adhere to the wells prior to imaging. The Zeiss Live Cell Observer system (AxioObserver Z1, Zeiss) was used to image the cells at 37°C with 5% CO_2_. Within each well, six positions around the center of the well were set up and imaged at ×5 magnification every 10 min from approximately 90 min post seeding until 10 h post-seeding. The images taken at 2 h post-seeding, for every hour until 10 h post-seeding, were analyzed using AngioTool [66]. AngioTool computes several morphological and spatial parameters including the explant area, vessel area, vessels as a percentage of area, number of junctions (branching points), junction density, average vessel length, total vessel length, the number of end points, and the lacunarity (describes the distribution of the gaps).

#### Statistical analysis

All statistical analyses were performed using Minitab 17 or GenStat 13.1 (VSN International Ltd). Mean values were calculated for each individual sample for each parameter investigated, and the normality of the distribution of the data was assessed by an Anderson-Darling test. If a *P* value of ≤0.05 was obtained, then the data were not considered to have a normal distribution. Log10 transformations were carried out to achieve normality of the distribution of the data where required. Outliers were tested for using a Grubbs outlier test and were excluded.

In experiments 1 and 2, fetal size was compared between the overall lightest fetus with the overall CTMLW fetus regardless of sex. Fetal sex was compared using samples from the lightest and CTMLW fetuses (GD30) of both sex (GD45, 60, and 90). Where data had a normal distribution, ANOVA for GD, fetal size, or sex was performed, with a block for gilt to account for the common maternal environment. A post hoc Tukey test was then performed. Where data did not have a normal distribution, Kruskal-Wallis and Mann-Whitney tests were performed where appropriate. Analyses for fetal size and sex were performed overall and within GD of interest. In experiment 3, for each parameter for each sample, the mean value from the 12 images was divided by the media only control. Where the data had a normal distribution, ANOVA was performed with a block for gilt. This was performed for the GD and size effects using the GD45 and 60 data overall, combining the data from all nine culture time points, and within each time point. On occasions when the data could not be transformed to achieve a normal distribution, Kruskal-Wallis tests were performed for the overall effect, with all time points combined, followed by a Mann-Whitney test within time point for GD, size, and sex.

In all experiments results were considered significant when *P* ≤ 0.05, trending towards significant when *P* was >0.05, <0.1, and not significant when *P* ≥ 0.1.

## Results

### Experiment 1: Analysis of placental vascularity by immunohistochemistry for CD31

The total number of blood vessels, percentage CD31 staining, internal and external blood vessel diameters, and blood vessel wall thickness at GD45 or 60 did not differ between the lightest and the CTMLW fetus (Supplementary Table S7).

The total number of blood vessels, percentage CD31 staining, internal and external blood vessel diameters, and blood vessel wall thickness at GD45 or 60 did not differ between placentas supplying fetuses of different sex at GD45 or 60 (Supplementary Table S7).

**Figure 1.**
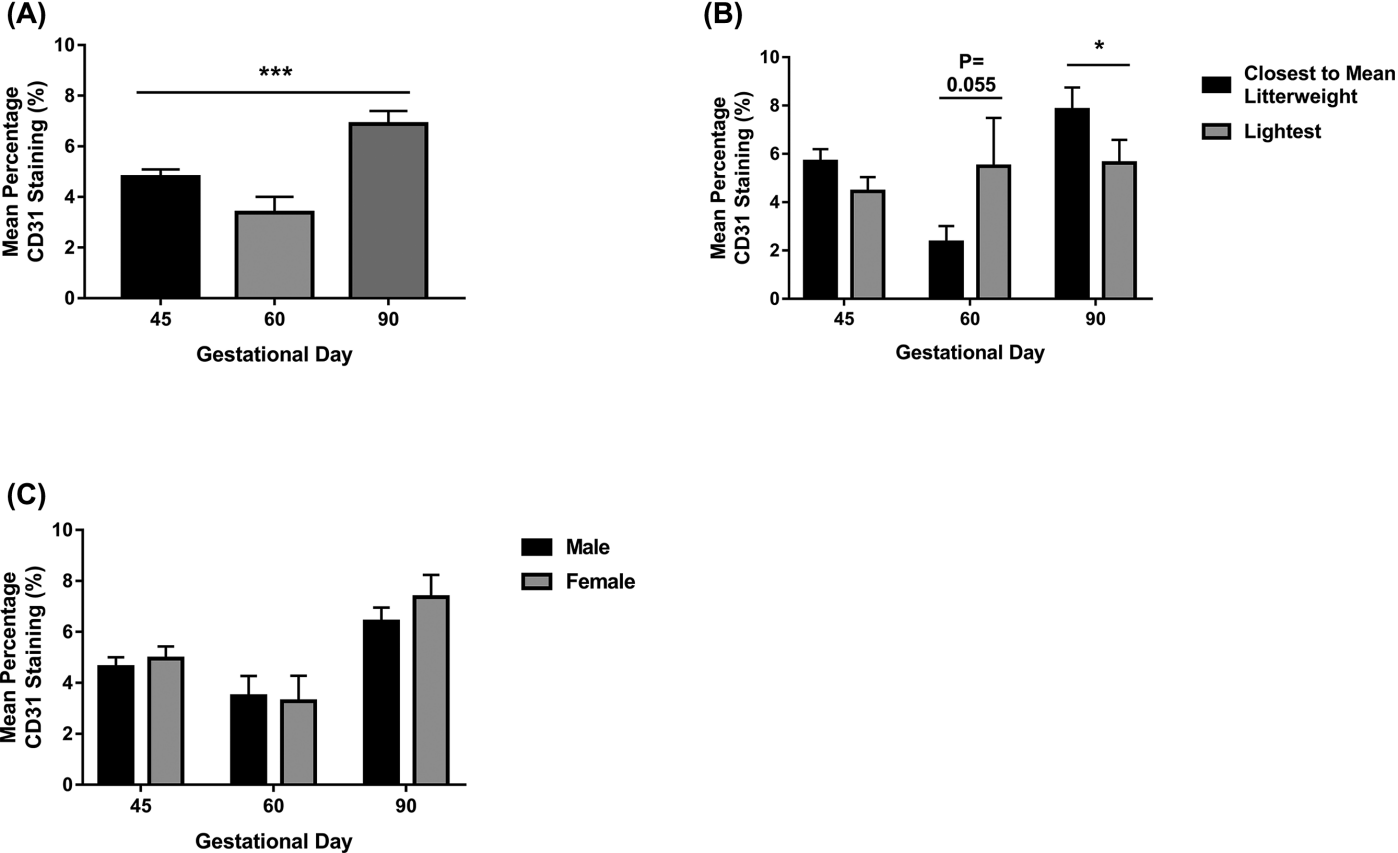
Fetal size is associated with CAM CD31 staining at GD60 and 90. The percentage CD31 staining of the CAM was investigated at GD45, 60, and 90 (A) (N = 20–28 placental samples per group). The association between fetal size (B; N = 5–8 samples per group) and fetal sex (N = 10–14 samples per group) and percentage CD31 staining of the CAM was investigated. Error bars represent SEM. *P ≤ 0.05. ***P ≤ 0.001.

An overall GD effect in CD31 staining was observed (*P* ≤ 0.001) (Figure 1A). Decreased CD31 CAM staining was observed in GD60 placental samples compared to GD45 placental samples (Figure 1A). CD31 CAM staining peaked at GD90. Fetal size was not associated with CD31 percentage staining in the CAM at GD45 (Figure 1B). Placentas supplying the lightest fetuses at GD60 had an increased percentage staining CD31 in the CAM compared to those supplying the CTMLW fetuses (*P* = 0.055). However, at GD90 placentas supplying the lightest fetuses had decreased CD31 staining in the CAM compared to those supplying the CTMLW fetuses (*P* = 0.05). No differences in the placental CAM CD31 staining were observed between placentas supplying fetuses of different sex at GD45, 60, or 90 (Figure 1C).

### Experiment 2: Analysis of candidate gene expression by qPCR

Temporal changes were observed in the mRNA expression of the candidate genes investigated (Supplementary Figure S1). *ACP5* (Supplementary Figure S1A), and *PTGFR* (*P* ≤ 0.001; Supplementary Figure S1E) had low expression at GD30 which increased at GD45 and remained elevated for the remainder of gestation. *CD31* (*P* ≤ 0.01 Supplementary Figure S1B), *HIF1A* (*P* ≤ 0.01; Supplementary Figure S1C), and *HPSE* (*P* ≤ 0.01; Supplementary Figure S1D) all had a similar temporal profile with consistent expression between GD30, 45, and 60 and increased expression at GD90. *VEGFA* expression increased between GD30 and 45, plateaued between GD45 and 60, and increased between GD60 and 90 (*P* ≤ 0.001; Supplementary Figure S1F).

**Figure 2.**
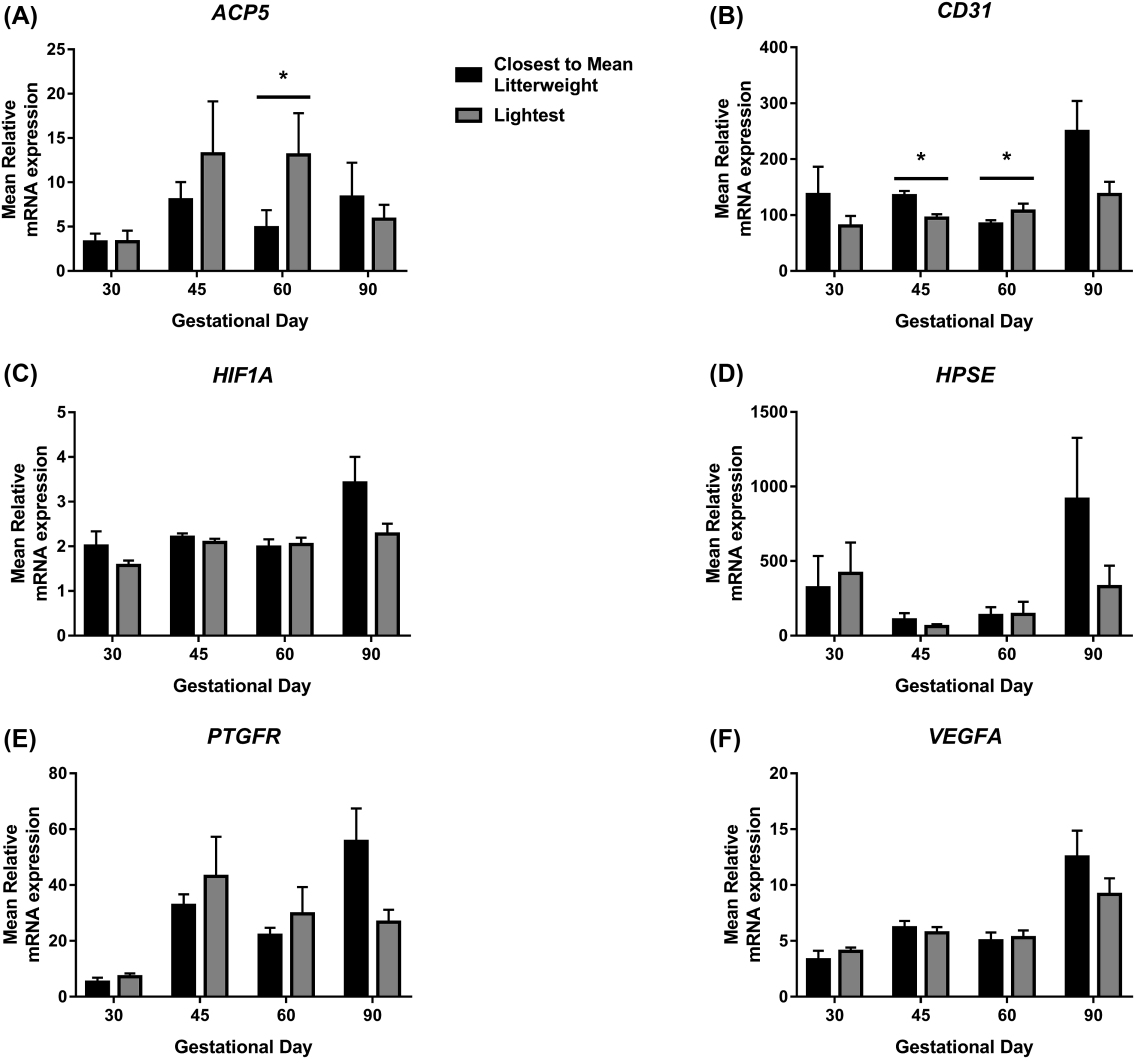
Fetal size is associated with altered placental *ACP5* and *CD31* expression. mRNA expression of *ACP5* (A), *CD31* (B), *HIF1A* (C), *HPSE* (D), *PTGFR* (E), and *VEGFA* (F) was quantified by qPCR in placental samples associated with the lightest and CTMLW fetuses at GD30, 45, 60, and 90. Mean values presented. N = 4–7 placental samples per group. Error bars represent SEM. *P ≤ 0.05.

*ACP5* expression was increased in placentas supplying the lightest fetuses compared to those supplying the CTMLW fetuses at GD60 (*P* ≤ 0.05; Figure 2A). The expression of *CD31* was decreased in placentas supplying the lightest fetuses compared to those supplying the CTMLW fetuses at GD45 (*P* ≤ 0.05; Figure 2B). The direction of this difference switched at GD60, with placentas supplying the lightest fetuses having increased *CD31* expression compared to those supplying the CTMLW fetuses (*P* ≤ 0.05). No significant association between fetal size and *HIF1A, HPSE, PTGFR*, or *VEGFA* expression was observed (Figure 2C, D, E, and F respectively).

No associations between fetal sex and placental mRNA expression were observed in *ACP5, CD31, HPSE, PTGFR*, or *VEGFA* expression (Supplementary Figure S2A, B, D, E, and F, respectively). At GD90, a trend towards increased *HIF1A* expression was observed in placental samples supplying female fetuses compared to those supplying male fetuses at GD90 (*P* = 0.073; Supplementary Figure S2C).

### Experiment 3: Analysis of in vitro endothelial cell branching in response to placental conditioned media

Following the culture period, Ki67 staining revealed that the explants were still proliferating (data not presented). It was demonstrated that the G-1410 endothelial cells express endothelial cell markers on both a protein and mRNA level (Supplementary Figure S3).

**Figure 3.**
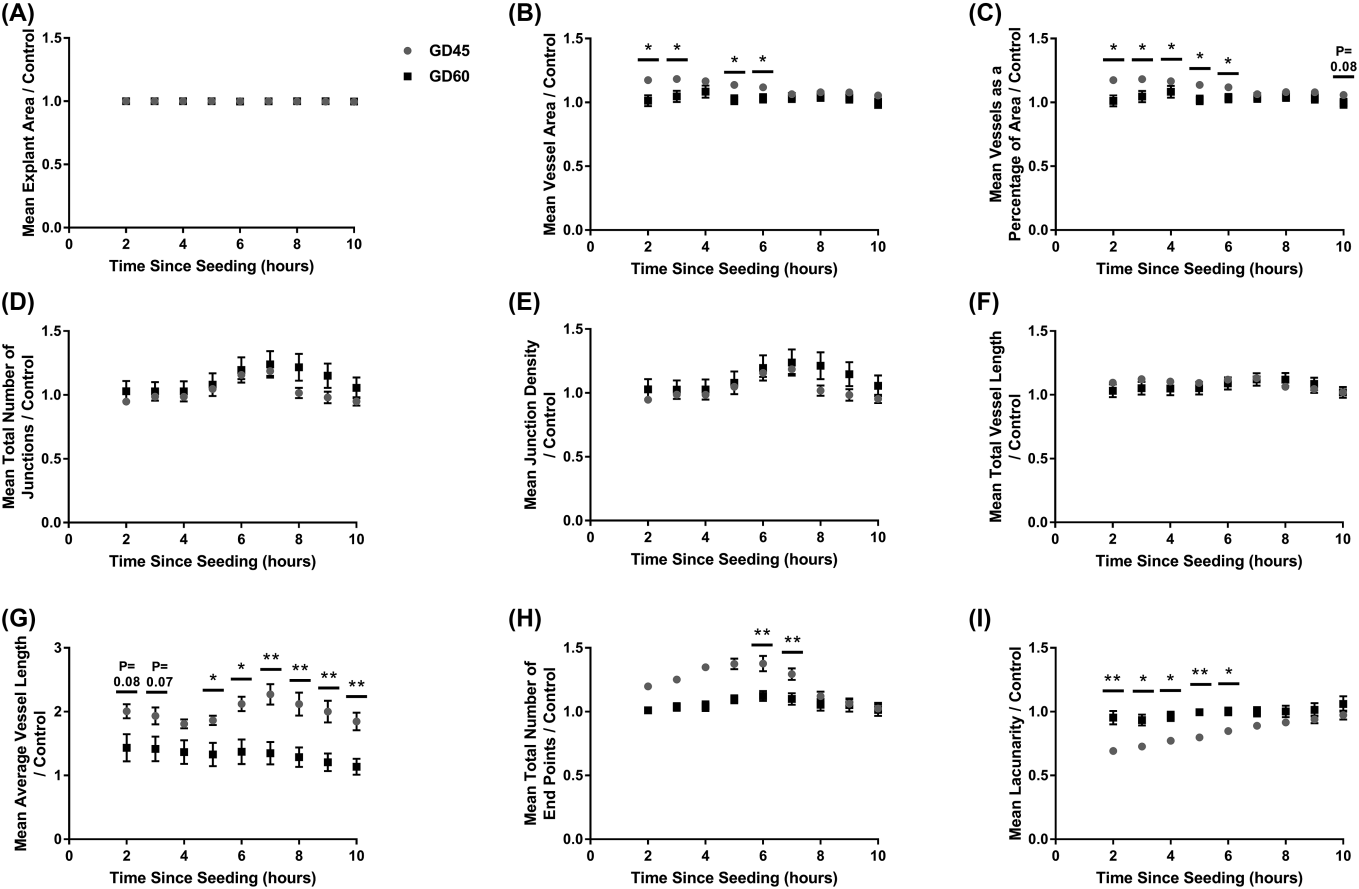
The GD of placental samples influences endothelial cell branching in vitro. Endothelial cells were cultured in conditioned media generated from culture of placental samples at GD45 and 60. The influence of the conditioned media on explant area (A), vessel area (B), vessels as a percentage of area (C), total number of junctions (D), junction density (E), total vessel length (F), average vessel length (G), total number of end points (H), and lacunarity (I) was investigated from 2–10 h post-seeding of the cells. Mean values presented for 6 and 12 GD45 and 60 placental samples, respectively. Error bars represent SEM. *P ≤ 0.05. **P ≤ 0.01.

No differences in the response of endothelial cells to the conditioned media from placentas from the two GD (45 and 60) were observed in explant area (Figure 3A), total number of junctions (Figure 3D), junction density (Figure 3E), or total vessel length (Figure 3F). The endothelial cells cultured with GD45 conditioned media had an increased vessel area at 2, 3, 5, and 6 h post-seeding (*P* ≤ 0.05; Figure 3B), and vessels as a percentage of the total area at 2–6 (*P* ≤ 0.05) and 8 h (*P* = 0.08) post-seeding compared to cells cultured with GD60 conditioned media. The average vessel length was increased throughout the culture period in response to culture with conditioned media from GD45 compared to the GD60 placentas (*P* = 0.08 at 2 h, *P* = 0.07 at 3 h, *P* ≤ 0.05 at 5 and 6 h, and *P* ≤ 0.01 at 7–10 h post-seeding; Figure 3G). An increase in the number of end points in response to the GD45 placental media was observed at 2–7 h post-seeding (significant at 6 and 7 h post-seeding (*P* ≤ 0.01); Figure 3H). An overall GD effect was observed when comparing the two GD over the course of the experiment (*P* ≤ 0.01; Figure 3I), with endothelial cells cultured with the GD45 conditioned media having decreased lacunarity compared with those that had been cultured with conditioned media from GD60 placentas. This was significant at 2–6 h post-seeding (*P* ≤ 0.01 at 2 and 5 h, *P* ≤ 0.05 at 3, 4, and 6 h post-seeding).

**Figure 4.**
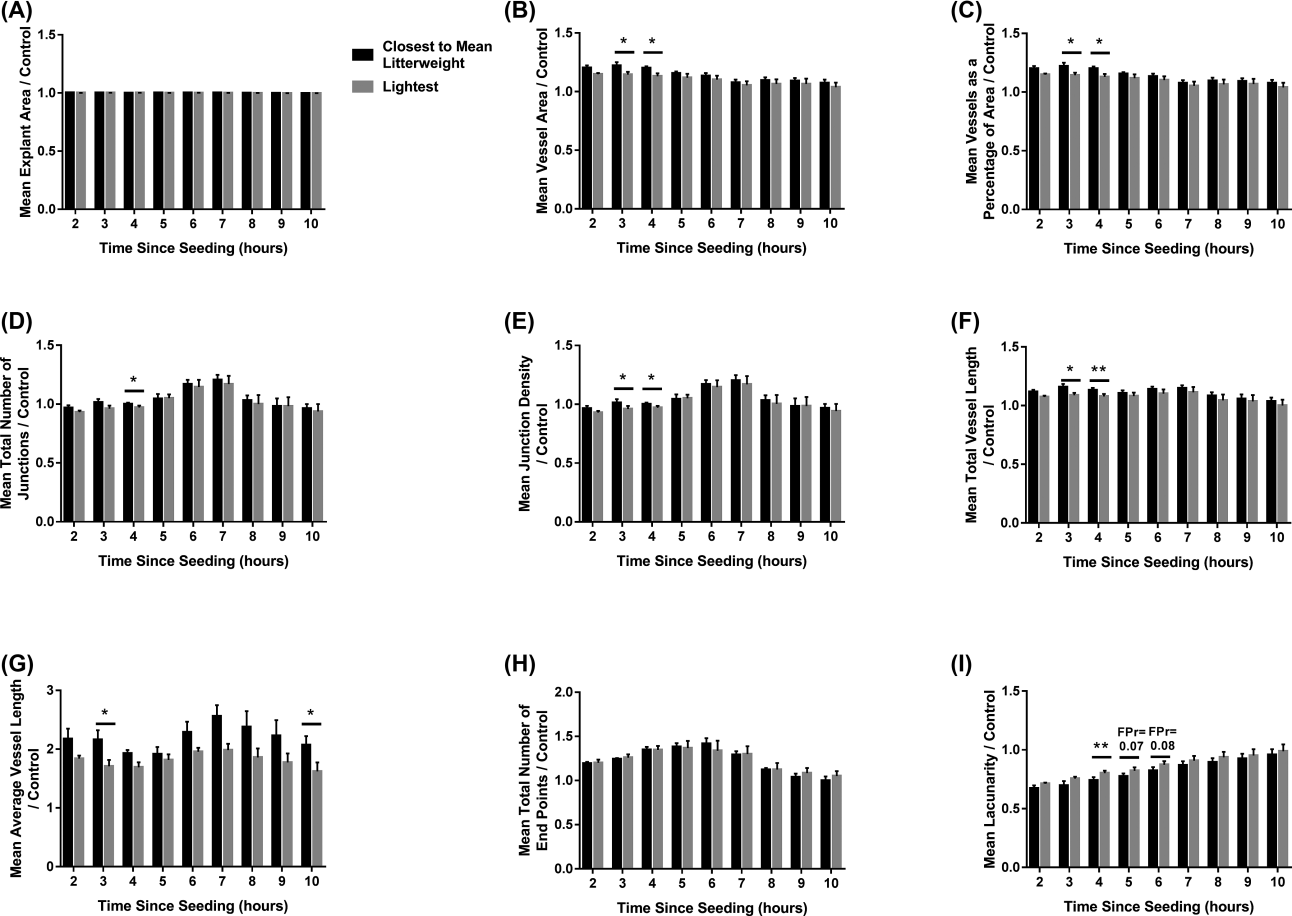
Endothelial cell branching is impaired in response to conditioned media from placentas supplying the lightest fetuses at GD45. Endothelial cells were cultured in conditioned media generated from culture of placental samples supplying the lightest and CTMLW fetuses at GD45. The influence of the conditioned media on explant area (A), vessel area (B), vessels as a percentage of area (C), total number of junctions (D), junction density (E), total vessel length (F), average vessel length (G), total number of end points (H), and lacunarity (I) was investigated from 2–10 h post-seeding of the cells. Mean values presented for three placental samples in each group. Error bars represent SEM. *P ≤ 0.05. **P ≤ 0.01.

Whilst no relationship between fetal size and explant area (Figure 4A) or total number of end points (Figure 4H) was observed, fetal size did influence the remaining parameters investigated. Endothelial cells cultured with conditioned media from the placentas supplying the lightest fetuses decreased the overall vessel area (*P* ≤ 0.01), compared to endothelial cells cultured with media from the CTMLW fetuses (Figure 4B; significant at 3 and 4 h post-seeding (*P* ≤ 0.05)). Endothelial cells cultured with conditioned media from the placentas supplying the lightest fetuses decreased vessels as a percentage of area with all time points investigated combined (*P* ≤ 0.01; Figure 4C). This decrease was significant within time point at 3 and 4 h post-seeding (*P* ≤ 0.05). Endothelial cells cultured with conditioned media from placentas supplying the lightest fetuses compared to the CTMLW fetuses had fewer junctions (Figure 4D) at 4 h post-seeding (*P* ≤ 0.05), and a lower density of the junctions (Figure 4E) at 3 and 4 h post-seeding (*P* ≤ 0.05). An overall decrease was observed in both total vessel length (Figure 4F; *P* ≤ 0.01) and average vessel length (Figure 4G; *P* ≤ 0.001) in response to conditioned media from the lightest compared to the CTMLW fetuses. This decrease was significant in total vessel length at 3 (*P* ≤ 0.05) and 4 h (*P* ≤ 0.01) and average vessel length at 3 h (*P* ≤ 0.05) post-seeding. Lacunarity was increased following culture with conditioned media from the lightest compared to the CTMLW fetuses at 4 (*P* ≤ 0.01), 5, and 6 (*P* = 0.07 and 0.08, respectively) h post-seeding (Figure 4I).

**Figure 5.**
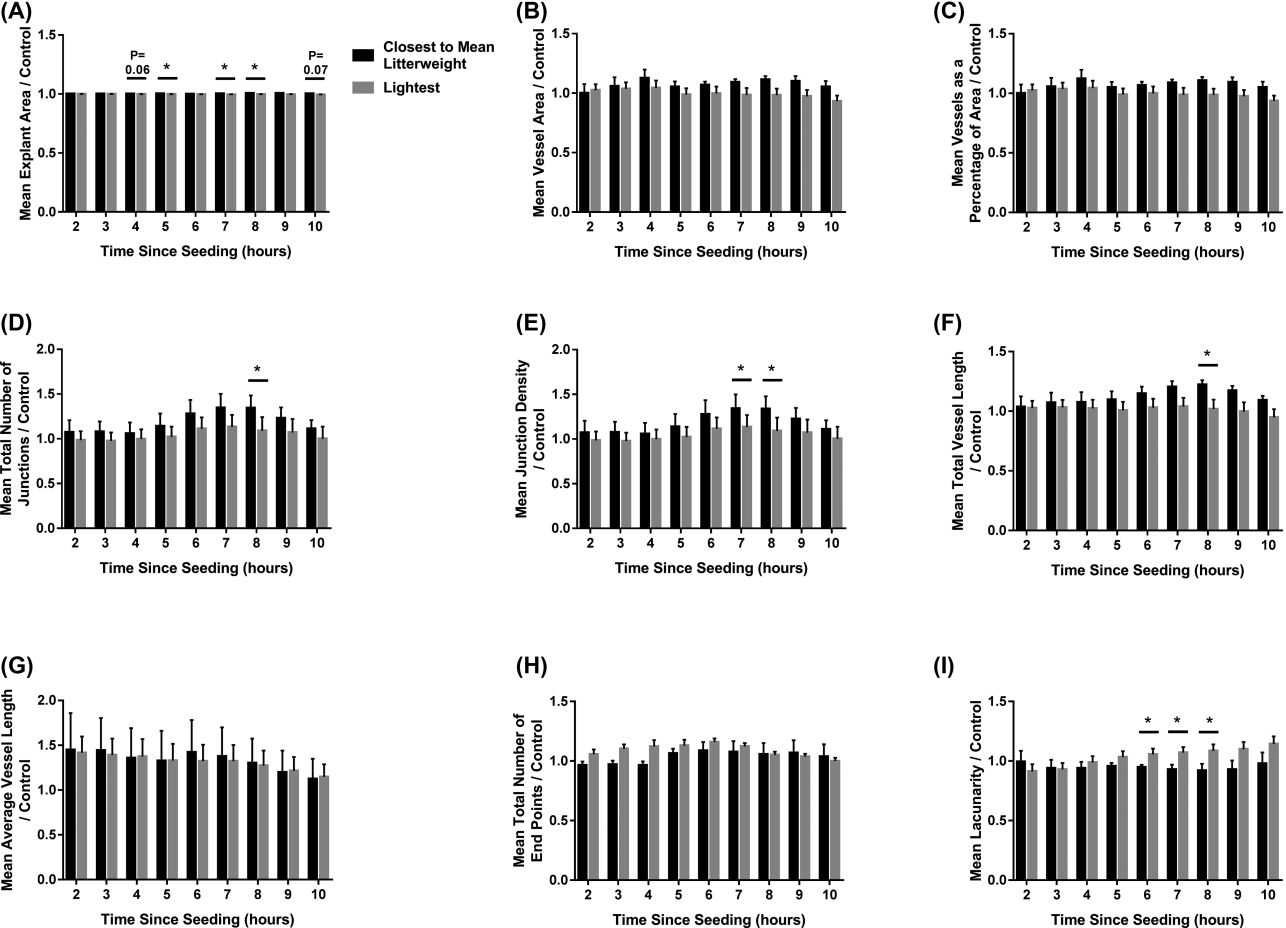
Endothelial cell branching impaired in response to conditioned media from placentas supplying the lightest fetuses at GD60. Endothelial cells were cultured in conditioned media generated from culture of placental samples supplying the lightest and CTMLW fetuses at GD60. The influence of the conditioned media on explant area (A), vessel area (B), vessels as a percentage of area (C), total number of junctions (D), junction density (E), total vessel length (F), average vessel length (G), total number of end points (H), and lacunarity (I) was investigated from 2–10 h post-seeding of the cells. Mean values presented for six placental samples in each group. Error bars represent SEM. *P ≤ 0.05. **P ≤ 0.01.

Overall size effects were observed, with a decrease in explant area (*P* ≤ 0.001; Figure 5A), vessel area (*P* ≤ 0.01; Figure 5B), vessels as a percentage of area (*P* ≤ 0.01; Figure 5C), the total number of junctions (*P* ≤ 0.001; Figure 5D), junction density (*P* ≤ 0.001; Figure 5E), and total vessel length (*P* ≤ 0.001; Figure 5F) observed when endothelial cells were cultured with conditioned media from the lightest compared to the CTMLW fetuses. An increase in the total number of end points (*P* ≤ 0.001, Figure 5H) and lacunarity (*P* ≤ 0.001, Figure 5I) was observed in endothelial cells cultured with conditioned media from the lightest compared to the CTMLW fetuses. Fetal size at GD60 was not associated with average vessel length (Figure 5G). The decrease in mean explant area was significant within time point at 4 (*P* = 0.06), 5, 7, 8 (all *P* ≤ 0.05), and 10 (*P* = 0.07) h post-seeding (Figure 5A). No differences were observed within time point for vessel area (Figure 5B), vessels as a percentage of area (Figure 5C), average vessel length (Figure 5G), or total number of end points (Figure 5H). The decrease in the total number of junctions in endothelial cells cultured with conditioned media from the lightest compared to the CTMLW fetuses was significant at 8 h post-seeding (*P* ≤ 0.05; Figure 5D). The observed decrease in junction density was significant at 7 and 8 h post-seeding (*P* ≤ 0.05; Figure 5E). Endothelial cells cultured with conditioned media from the lightest fetuses resulted in decreased total vessel length at 8 h post-seeding (*P* ≤ 0.05; Figure 5F) compared to cells cultured with conditioned media from the CTMLW fetuses. The increase in lacunarity observed was significant at 6–8 h (*P* ≤ 0.05; Figure 5I) post-seeding.

**Figure 6.**
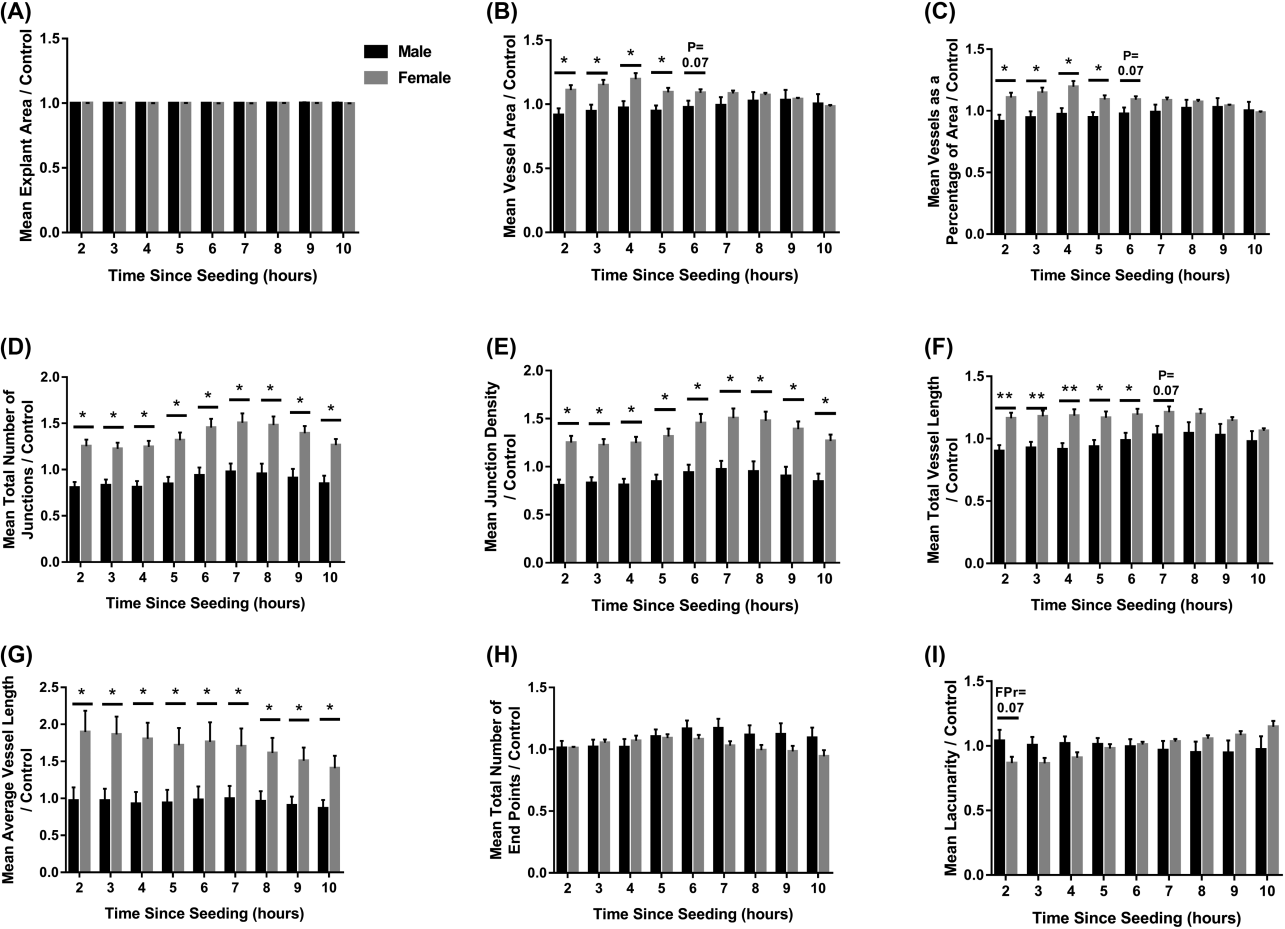
Fetal sex influences endothelial cell branching in response to placental conditioned media at GD60. Endothelial cells were cultured in conditioned media generated from culture of placental samples supplying male and female fetuses at GD60. The influence of the conditioned media on explant area (A), vessel area (B), vessels as a percentage of area (C), total number of junctions (D), junction density (E), total vessel length (F), average vessel length (G), total number of end points (H), and lacunarity (I) was investigated from 2–10 h post-seeding of the cells. Mean values presented for six placental samples in each group. Error bars represent SEM. *P ≤ 0.05. **P ≤ 0.01.

An overall sex effect was observed for explant area (*P* ≤ 0.05; Figure 6A), vessel area (*P* ≤ 0.001; Figure 6B), vessels as a percentage of area (*P* ≤ 0.001; Figure 6C), total number of junctions (*P* ≤ 0.05; Figure 6D), junction density (*P* ≤ 0.05; Figure 6E), total vessel length (*P* ≤ 0.001; Figure 6F), and average vessel length (*P* ≤ 0.001; Figure 6G), with conditioned media from placentas supplying female fetuses having an increased effect on endothelial cell branching compared to those supplying male fetuses. No differences were observed in explant area (Figure 6A) or total number of end points (Figure 6H) in relation to fetal sex. Mean vessel area and vessels as a percentage of area were both increased in response to conditioned media from placentas supplying female fetuses at 2–5 (all *P* ≤ 0.05; both parameters) and 6 (*P* = 0.07; both parameters) h post-seeding (Figure 6B and C), when compared with conditioned media from placentas supplying male fetuses. The total number of junctions (Figure 6D), junction density (Figure 6E), and average vessel length (Figure 6G) were increased in response to conditioned media from placentas supplying female fetuses compared with conditioned media from placentas supplying male fetuses at all time points investigated (all *P* ≤ 0.05). Total vessel length increased in response to conditioned media from placentas supplying female fetuses at 2, 3, 4 (all *P* ≤ 0.01), 5, 6 (both *P* ≤ 0.05), and 7 (*P* = 0.07) h post-seeding (Figure 6F) compared to those supplying male fetuses. A trend towards decreased lacunarity (*P* = 0.07) was observed in response to conditioned media from placentas supplying female fetuses compared to male fetuses at 2 h post-seeding.

## Discussion

Improved understanding of the mechanisms governing fetal growth is essential to reduce the prevalence of low birthweight piglets and so improve the efficiency of the pig production industry. The present study is part of a larger study investigating the effects of fetal size and sex on the feto–maternal interface at five key stages of gestation. This study utilized complementary analytical approaches (immunohistochemistry, qPCR, and endothelial cell branching assays) to gain a more integrated understanding of the relationship between fetal size and sex, and placental vascularity throughout gestation. The results of this study have demonstrated a temporal relationship between fetal size and placental gene expression throughout gestation. Dynamic changes in gene expression between GD45 and 60 were observed suggesting striking changes in placental vascularity during this period of gestation. Conditioned media from placental samples supplying fetuses of different size, and more intriguingly sex, were shown to have differential abilities to induce endothelial cell branching in vitro.

It is thought that there are two phases of angiogenesis in the pregnant pig uterus, the first around the time of conceptus attachment (GD13–18) [2], followed by a second phase of angiogenesis in mid-gestation until term to increase the availability of nutrients to the exponentially growing fetus [3]. The decreased branching ability in response to placental conditioned media at GD60 could reflect that at this GD placental growth is no longer exponential and the placenta had already initiated the second wave of angiogenesis. Extensive remodeling of the placenta must occur in mid-gestation to meet the increasing requirements of the exponentially growing fetus [11]. The dynamic changes in placental structure and function in mid-gestation were reflected in the striking changes in gene expression observed between GD45 and 60.

Blomberg et al. [67] demonstrated that endothelial nitric oxide synthase (*eNOS*) expression is increased in IUGR placentas at GD50 compared to control placentas. Endothelial NOS is known to induce angiogenesis [68, 69] which may suggest a mechanism whereby at this stage of gestation the placentas supplying IUGR fetuses could compensate for their decreased nutrient supply. The results presented in this study provide evidence to support the presence of a compensation mechanism developing around this stage of gestation. While the influence of fetal size on in vitro branching ability when cultured with GD60 conditioned media was not as pronounced as at GD45 it was still present. This may indicate that the attempt of the placentas supplying the lightest fetuses to “catch up” is ultimately not going to be successful in rescuing the growth restriction phenotype.

An association between fetal size and the percentage CD31 staining of the CAM was observed and the qPCR data reinforced this difference. It is thought that to compensate for having smaller placentas, the placentas supplying small fetuses increase the width of the bilayers more quickly to form larger folds, thereby providing a larger area for exchange and improving the efficiency of the placenta. Vallet and Freking [70] have previously suggested that the folded bilayer is wider in placentas supplying the smallest fetuses at GD65, 85, and 105 in placentas compared to placentas supplying the largest fetuses. They also demonstrated that the stroma above the CAM decreased in size at a faster rate in the placentas supplying the smallest fetuses compared to the largest fetuses. In a follow-up study, Vallet et al. demonstrated that placentas supplying small fetuses have increased hyaluronoglucosaminidase mRNA expression and increased hyaluronan synthesis compared to those supplying the largest fetuses [71]. Considering this, it is believed that the increased remodeling of the porcine feto–maternal interface supplying the smallest fetuses could act as a compensation mechanism to attempt to ensure adequate nutrient transfer to the smaller fetuses. The results presented in this study suggest that it is not only the width and remodeling of the bilayer, but also the vascularity of the bilayer which may act to attempt to rescue the IUGR phenotype.

The present study observed that the direction of differences in placental gene expression between the feto–placental units of the lightest fetuses compared with the CTMLW fetuses was reversed between GD45 and 60. This may indicate that the tissues associated with small fetuses are attempting to rescue fetal growth by transcriptionally altering the expression of genes important in regulating placental and fetal development. *eNOS* expression has previously been shown to be increased in IUGR placentas at GD50 compared to control placentas [67], providing further evidence for the potential presence of a mechanism in placentas supplying small fetuses to compensate for having a disadvantaged blood supply. The dynamic changes in the direction of differences in gene expression in placentas associated with the lightest fetuses observed between GD45 and 60 suggest that the relationship between the fetus and the placenta is highly dynamic at this stage of gestation. It was hypothesized that fetal sex would influence placental structure, gene expression, and angiogenic potential in the pig. In the present study, fetal sex was not found to be associated with the mRNA expression of the genes investigated, or the structure of the placenta. However, it was associated with endothelial cell branching in vitro, with conditioned media from placentas supplying females increasing endothelial cell branching in vitro compared to those supplying males. This may indicate that the expression of other components of the angiogenesis pathways is influenced by fetal sex. It is also important to note that the in vitro branching assays do not fully reflect the complexity of the in vivo functions of the placenta; therefore, the significance of the fetal sex result should not be overinterpreted.

Whilst the knowledge of sexual dimorphism in placental development has been described in humans [23] and rats [72], the knowledge of sexual dimorphism in placental development in large animal species is poorly understood. In bovine GD19 conceptuses, it has been reported that >5000 transcripts are differentially expressed in conceptuses of different sex [73], highlighting the early development of sexual dimorphism in fetal development. Considering this, and the demonstration that porcine male conceptuses have an increased growth rate compared to female conceptuses from GD10 [74], it would be highly likely that sexual dimorphism in conceptus gene expression/protein secretion would be observed during implantation in the pig. We propose that this not only results in sexual dimorphism in placental development and function, but also that these differences may result in the uterus being “primed” differently for fetuses of different sex.

## Conclusions

This study has demonstrated associations between fetal size and placental vasculature, gene expression, and the in vitro angiogenic potential of the porcine placenta, which are influenced by gestational day. Intriguingly, fetal sex was associated with the angiogenic potential of placental samples in vitro, with conditioned media from placentas supplying male fetuses impairing endothelial cell branching compared to female fetuses. Changes in the direction of differences in gene expression in placentas associated with the lightest fetuses observed between GD45 and 60 suggest that the relationship between the fetus and the placenta is highly dynamic at this stage of gestation and warrants further investigation.

## Supplementary data

**Supplementary Figure S1.** Gestational changes were observed in the placental expression of all candidate genes investigated. mRNA expression of *ACP5* (A), *CD31* (B), *HIF1A* (C), *HPSE* (D), *PTGFR* (E), and *VEGFA* (F) was quantified by qPCR in placental samples at GD30, 45, 60, and 90. Mean values are presented. Error bars represent SEM. Letters indicate that group means differ from one another, tested by post-hoc Tukey.

**Supplementary Figure S2.** No statistically significant associations between fetal sex and placental mRNA expression were observed. mRNA expression of *ACP5* (A), *CD31* (B), *HIF1A* (C), *HPSE* (D), *PTGFR* (E), and *VEGFA* (F) was quantified by qPCR in placental samples associated with fetuses of different sex at GD30, 45, 60, and 90. Error bars represent SEM.

**Supplementary Figure S3.** G-1410 cells express endothelial cell markers. (A) Immunocytochemistry was performed for the endothelial cell markers CD31 and vWF. First column: Cells were stained with an equivalent total protein concentration of RIgG to that of the primary antibody. Second column: cells were stained with only the secondary antibody. Third column: positive staining was observed for CD31 (top panel) and vWF (bottom panel). Scale bar represents 20 μm. The cells were imaged using LSM 710 confocal microscope at ×40 magnification (Zeiss). (B) RNA was extracted from the endothelial cells and a PCR was performed for (1) *B2M1* (reference gene control), (2) *CD31*, two isoforms of VEGFA (3) *VEGF120* and (4) *VEGF164*, and (5) *VEGFR1*. Top panel: PCR with template cDNA. Bottom panel: PCR with NRT control. Electrophoresis performed using a TAE gel stained with SYBR Safe. PCR markers used as ladder.

**Supplementary Table S1.** Summary of litter characteristics of the gilts used.

**Supplementary Table S2.** Antibodies used.

**Supplementary Table S3.** Summary of RNA quality assessment.

**Supplementary Table S4.** Primer sequence details for qPCRs.

**Supplementary Table S5.** Quantitative polymerase chain reaction calibration curve data.

**Supplementary Table S6.** Primer sequences used for amplification of endothelial cell cDNA by PCR.

**Supplementary Table S7.** CD31 stained placental stromal immunohistochemical analysis.

Supplemental Tables and Figures

## Notes

**Conference Presentation:** Presented in part (as a poster) at the following conferences: Little Embryos Do Make Big Decisions Symposium, Society for Reproduction and Fertility Annual Conference 2016 and the 18th International Conference on Animal Reproduction. Orally presented in part at the following conferences: Society for Reproduction and Fertility Annual Conference 2015 and Fertility 2018.
