# Supplementary material for: Associations between fetal size, sex and placental angiogenesis in the pig
Source: Biol Reprod. 2018 Aug 18;100(1):239–52. doi: 10.1093/biolre/ioy184 (PMC6335214; doi:10.1093/biolre/ioy184)
Supplement: Supplemental Tables and Figures [file ioy184_supplemental_tables_and_figures.zip › Supplementary Table 1.docx]

**Supplementary Table 1: Summary of Litter Characteristics of the Gilts Used**

| **Parameter** | **GD30 (n=6)**  **(PMSG=3/6)** | | **GD45 (n=6)**  **(PMSG=1/6)** | | **GD60 (n=11)**  **(PMSG=4/11)** | | **GD90 (n=8)**  **(PMSG=3/8)** | |
| --- | --- | --- | --- | --- | --- | --- | --- | --- |
|  | **Mean ± S.E.M.** | **Range** | **Mean ± S.E.M.** | **Range** | **Mean ± S.E.M.** | **Range** | **Mean ± S.E.M.** | **Range** |
| **Gestational Day (GD)** | 30 | n/a | 45 ± 0.516 | 43 - 46 | 60.272 ± 0.304 | 58 - 62 | 90.375 ± 0.324 | 89 - 92 |
| **Ovulation Rate** | 18.833 ± 1.701 | 13 - 25 | 21.5 ± 2.814 | 14 - 33 | 21.364 ± 2.905 | 14 - 49 | 22.375 ± 2.345 | 15 - 37 |
| **Litter Size** | 11.5 ± 2.513 | 6-15 | 16.5 ± 1.455 | 12 - 20 | 14 ± 1.095 | 9 - 19 | 14.375 ± 0.844 | 13 - 20 |
| **Prenatal Survival (%)** | 60.315 ± 10.837 | 35.294 - 100 | 80.382 ± 7.599 | 57.143 - 93.333 | 70.227 ± 5.706 | 36.735 - 86.666 | 67.044 ± 5.215 | 54.054 - 93.333 |
| **Total Litter Weight (TLW) (g)** | 16.949 ± 2.916 | 9.022 - 28.701 | 343.110 ± 36.872 | 191.11 - 465.24 | 1647.577 ± 77.480 | 1305.910 - 2137.160 | 8854.700 ± 709.521 | 6467.490 - 13151.890 |
| **Mean Litter Weight (MLW) (g)** | 1.553 ± 0.112 | 1.304 - 2.061 | 20.815 ± 1.603 | 15.923 - 25.780 | 121.871 ± 6.102 | 89.186 - 150.056 | 635.359 ± 23.984 | 539.381 - 759.266 |
| **Mean Within-Litter SD in Fetal Weight (g)** | 0.194 ± 0.024 | 0.104 - 0.261 | 1.939 ± 0.320 | 0.927 -  3.038 | 12.150 ± 0.853 | 7.367 - 17.296 | 123.723 ± 16.826 | 40.253 - 205.243 |
| **Weight CTMLW Fetuses (g)** | 1.592 ± 0.101 | 1.334 - 2.058 | 20.793 ± 1.577 | 15.920 - 25.660 | 122.225 ± 6.360 | 86.96 - 150.24 | 620.125 ± 24.866 | 544.88 - 756.75 |
| **Weight Lightest Fetuses (g)** | 1.239 ± 0.103 | 0.901 - 1.661 | 17.827 ± 1.849 | 11.240 - 23.890 | 97.003 ± 8.267 | 45.76 - 128.71 | 377.519 ± 36.294 | 532.75 -248.95 |
| **Percentage Males in Litter (%)** | 65.387 ± 5.356 | 50 - 83.333 | 47.009 ± 9.177 | 30 - 80 | 51.794 ± 3.864 | 33.333 - 72.222 | 54.528 ± 5.310 | 33.333 - 76.923 |

Abbreviations Used: SD = Standard Deviation. Ovulation Rate = Number of *Corpora Lutea* Present. Prenatal Survival (%) = (Number of Live Fetuses/Ovulation Rate) x 100. CTMLW = Closest to Mean Litter Weight fetus. PMSG=Pregnant Mare Serum Gonadotropin. n/a = not applicable.
