# Supplementary material for: Associations between fetal size, sex and placental angiogenesis in the pig
Source: Biol Reprod. 2018 Aug 18;100(1):239–52. doi: 10.1093/biolre/ioy184 (PMC6335214; doi:10.1093/biolre/ioy184)
Supplement: Supplemental Tables and Figures [file ioy184_supplemental_tables_and_figures.zip › Supplementary Table 2.docx]

**Supplementary Table 2: Antibodies Used**

| **Antibody** | | **Company** | **Product Number** | **Research Resource Identifier** | **Application Used** | **Species Raised In** | **Dilution Used** |
| --- | --- | --- | --- | --- | --- | --- | --- |
| Primary | CD31 | Abcam | ab28364 | AB_726362 | IHC/IC | Rabbit | 1:100/1:20 |
| Primary | Ki67 | Abcam | ab15580 | AB_443209 | IHC | Rabbit | 1:200 |
| Primary | vWF | Abcam | ab6994 | AB_305689 | IC | Rabbit | 1:400 |
| Secondary | Alexa Fluor 488 goat anti-rabbit (H+L) | ThermoFisher | A-11008 | AB_143165 | IC | Goat | 1:1000 |
| Secondary | Biotinylated anti-rabbit IgG | Vector Laboratories | PK6101 | AB_2336820 | IHC | Goat | 1:200 |

Abbreviations used: IHC=Immunohistochemistry; IC=Immunocytochemistry; CD31=Platelet and Endothelial Cell Adhesion Molecule 1; vWF=von Willebrand Factor.
