# Supplementary material for: Associations between fetal size, sex and placental angiogenesis in the pig
Source: Biol Reprod. 2018 Aug 18;100(1):239–52. doi: 10.1093/biolre/ioy184 (PMC6335214; doi:10.1093/biolre/ioy184)
Supplement: Supplemental Tables and Figures [file ioy184_supplemental_tables_and_figures.zip › Supplementary Table 3.docx]

| **GD** | **260/280** | **RINe** | |
| --- | --- | --- | --- |
|  | **Mean** | **Mean** | **Range** |
| **30** | 2.04 | 7.83 | 7.2-9.0 |
| **45** | 2.08 | 7.12 | 6.5-8.5 |
| **60** | 2.02 | 7.34 | 6.9-7.8 |
| **90** | 2.04 | 7.04 | 6.5-8.0 |

**Supplementary Table 3: Summary of RNA Quality Assessment**

GD=Gestational Day. 260/280 ratio generated spectrophotometrically using the Nanodrop ND-1000 (Labtech International Ltd., Heathfield, U.K.). RINe=RNA integrity number equivalent generated using the Tapestation 2200 (Agilent Technologies, Edinburgh, U.K.).
