# Supplementary material for: Associations between fetal size, sex and placental angiogenesis in the pig
Source: Biol Reprod. 2018 Aug 18;100(1):239–52. doi: 10.1093/biolre/ioy184 (PMC6335214; doi:10.1093/biolre/ioy184)
Supplement: Supplemental Tables and Figures [file ioy184_supplemental_tables_and_figures.zip › Supplementary Table 4.docx]

**Supplementary Table 4: Primer Sequence Details for qPCRs**

| **Gene Symbol** | **Gene Name** | **Accession Number** | **Primer Sequence (5’🡪 3’)** | | **Tm (^o^C)** | **Amplicon Size** | **Reference** |
| --- | --- | --- | --- | --- | --- | --- | --- |
| *ACP5* | Uteroferrin | NM_214209.1 | Fwd | GCAGCCAAGGAGGACTATGT | 60 | 130 | Hernandez *et al*, 2013 |
|  |  |  | Rev | GGTAGGCAGTGACCTTGTGT |  |  |  |
| *CD31* | Platelet And Endothelial Cell Adhesion Molecule 1 | NM_213907.1 | Fwd | CCGAGGTCTGGGAACAAAGG | 60 | 98 | n/a |
|  |  |  | Rev | AGCCTTCCGTTCTAGAATATCTGTT |  |  |  |
| *HIF1A* | Hypoxia Inducible Factor 1 Alpha Subunit | NM_001123124 | Fwd | CCATGCCCCAGATTCAAGAT | 60 | 64 | Oliver *et al*, 2011 |
|  |  |  | Rev | GGTGAACTCTGTCTAGTGCTTCCA |  |  |  |
| *HPSE* | Heparanase | NM_001146130.2 | Fwd | CAGACCCCACAAGAAGGTGT | 60 | 170 | Miles *et al*, 2009 |
|  |  |  | Rev | GTTCCAGCTCCAAAGAGCAC |  |  |  |
| *PTGFR* | Prostaglandin F2 alpha Receptor | NM_214059 | Fwd | TCAGCAGCACAGACAAGG | 60 | 151 | Kaczynski and Waclawik, 2013 |
|  |  |  | Rev | TTCACAGGCATCCAGATAATC |  |  |  |
| *VEGFA* | Vascular Endothelial Growth Factor A | NM_214084 | Fwd | GCCCACTGAGGAGTTCAACATC | 60 | 59 | Oliver *et al*, 2011 |
|  |  |  | Rev | GGCCTTGGTGAGGTTTGATC |  |  |  |
| *HPRT1* | Hypoxanthine phosphoribosyl-transferase 1 | DQ845175 | Fwd | GGACTTGAATCATGTTTGTG | 60 | 91 | Nygard *et al*, 2007 |
|  |  |  | Rev | CAGATGTTTCCAAACTCAAC |  |  |  |
| *TBP1* | TATA box binding protein | DQ845178 | Fwd | AACAGTTCAGTAGTTATGAGCCAGA | 60 | 153 | Nygard *et al*, 2007 |
|  |  |  | Rev | AGATGTTCTCAAACGCTTCG |  |  |  |

Abbreviations: Fwd = Forward Primer. Rev = Reverse Primer. Tm=Primer Melting Temperature.
