# Supplementary material for: Associations between fetal size, sex and placental angiogenesis in the pig
Source: Biol Reprod. 2018 Aug 18;100(1):239–52. doi: 10.1093/biolre/ioy184 (PMC6335214; doi:10.1093/biolre/ioy184)
Supplement: Supplemental Tables and Figures [file ioy184_supplemental_tables_and_figures.zip › Supplementary Table 5.docx]

Supplementary Table 5: Quantitative polymerase chain reaction calibration curve data.

| **Gene** | **Slope** | **Intercept** | **Amplification Efficiency (%)** | **RSq** |
| --- | --- | --- | --- | --- |
| *ACP5* | -3.451 | 23.705 | 94.9 | 0.997 |
| *CD31* | -3.390 | 24.097 | 97.2 | 0.999 |
| *HIF1A* | -3.375 | 24.911 | 97.8 | 0.994 |
| *HPSE* | -3.088 | 26.124 | 107.7 | 0.992 |
| *PTGFR* | -3.275 | 24.062 | 102 | 0.991 |
| *VEGFA* | -3.222 | 24.462 | 104.4 | 0.992 |
| *HPRT1* | -3.402 | 27.002 | 96.8 | 0.991 |
| *TBP1* | -3.254 | 27.211 | 102.9 | 0.990 |

Gene abbreviations: *ACP5*=Uteroferrin, *CD31*=Platelet and Endothelial Cell Adhesion Molecule 1, *HIF1A*=Hypoxia Inducible Factor 1 Alpha Subunit, *HPSE*=Heparanase, *PTGFR*=Prostaglandin F2α Receptor, *VEGFA*=Vascular Endothelial Growth Factor A, *HPRT1*=Hypoxanthine phosphoribosyl-transferase 1, and *TBP1*=TATA box binding protein 1.
