# Supplementary material for: Associations between fetal size, sex and placental angiogenesis in the pig
Source: Biol Reprod. 2018 Aug 18;100(1):239–52. doi: 10.1093/biolre/ioy184 (PMC6335214; doi:10.1093/biolre/ioy184)
Supplement: Supplemental Tables and Figures [file ioy184_supplemental_tables_and_figures.zip › Supplementary Table 6.docx]

Supplementary Table 6: Primer Sequences Used for Amplification of Endothelial Cell cDNA by PCR.

| **Gene Symbol** | **Gene Name** | **Accession Number** | **Primer Sequence (5’🡪 3’)** | | **Amplicon Size** | **Tm (^o^C)** | **Reference** |
| --- | --- | --- | --- | --- | --- | --- | --- |
| *B2M1* | Beta-2-microglobulin | DQ845172 | Fwd | CAAGATAGTTAAGTGGGATCGAGAC | 161 | 60 | Nygard *et al*, 2007 |
|  |  |  | Rev | TGGTAACATCAATACGATTTCTGA |  |  |  |
| *CD31* | Platelet and Endothelial Cell Adhesion Molecule 1 | NM213907.1 | Fwd | CCGAGGTCTGGGAACAAAGG | 98 | 60 | n/a |
|  |  |  | Rev | AGCCTTCCGTTCTAGAATATCTGTT |  |  |  |
| *VEGF120* | Vascular Endothelial Growth Factor (Splice Variants) | XM013977975.1 | Fwd | AAGGCCAGCACATAGGAGAG | 101 | 60 | Chrusciel *et al*, 2011 |
|  |  |  | Rev | CCTCGGCTTGTCACATTTTT |  |  |  |
| *VEGF164* |  | NM214084.1 | Fwd | GAGGCAAGAAAATCCCTGTG | 150 | 60 | Chrusciel *et al*, 2011 |
|  |  |  | Rev | TCACATCTGCAAGTACGTTCG |  |  |  |
| *VEGFR1* | VEGF Receptor 1 | XM021065524.1 | Fwd | CACCCCGGAAATCTATCAGATC | 180 | 60 | Chrusciel *et al*, 2011 |
|  |  |  | Rev | GAGTACGTGAAGCCGCTGTTG |  |  |  |

This table summarises the gene abbreviation and name, primer sequences, amplicon size, accession number, primer melting temperature (Tm) and the source where the primer sequence tested were obtained from. Fwd=Forward Primer. Rev=Reverse Primer.
