# Supplementary material for: Associations between fetal size, sex and placental angiogenesis in the pig
Source: Biol Reprod. 2018 Aug 18;100(1):239–52. doi: 10.1093/biolre/ioy184 (PMC6335214; doi:10.1093/biolre/ioy184)
Supplement: Supplemental Tables and Figures [file ioy184_supplemental_tables_and_figures.zip › Supplementary Table 7.docx]

**Supplementary Table 7: CD31 Stained Placental Stromal Immunohistochemical Analysis**

| **Parameter** | **GD45 (mean±S.E.M; (number of fetuses))** | | | | **GD60 (mean±S.E.M; (number of fetuses))** | | | |
| --- | --- | --- | --- | --- | --- | --- | --- | --- |
|  | **CTMLW** | **Lightest** | **Male** | **Female** | **CTMLW** | **Lightest** | **Male** | **Female** |
| **Mean Total BV Number** | 30.065 ± 5.481 (6) | 37.083 ± 5.356 (4) | 32.944 ± 4.798 (11) | 31.947 ± 4.449 (11) | 54.196 ± 9.649 (7) | 50.429 ± 9.180 (7) | 50.804 ± 4.766 (14) | 46.741 ± 5.767 (14) |
| **Mean Percentage CD31 Staining (%)** | 0.977 ± 0.135 (6) | 0.948 ± 0.170 (4) | 0.875 ± 0.071 (10) | 1.040 ± 0.095 (11) | 0.470 ± 0.210 (7) | 0.576 ± 0.159 (7) | 0.464 ± 0.111 (14) | 0.449 ± 0.091 (14) |
| **Mean Internal BV Diameter (µm)** | 9.587 ± 2.685 (6) | 6.080 ± 0.879 (4) | 10.861 ± 3.151 (11) | 9.236 ± 1.609 (11) | 15.290 ± 3.159 (7) | 12.413 ± 1.624 (7) | 13.874 ± 1.300 (14) | 15.115 ± 2.135 (14) |
| **Mean External BV Diameter (µm)** | 18.983 ± 3.394 (6) | 13.043 ± 1.014 (4) | 21.103 ± 4.937(11) | 18.257 ± 2.212(11) | 22.615 ± 2.301 (6) | 25.306 ± 2.819 (7) | 28.238 ± 2.076 (14) | 25.507 ± 2.610 (13) |
| **Mean BV Wall Thickness (µm)** | 9.395 ± 0.858 (6) | 6.963 ± 0.278 (4) | 10.242 ± 2.142 (11) | 9.021 ± 0.713 (11) | 12.411 ± 2.347 (7) | 12.893 ± 1.592 (7) | 14.364 ± 1.156 (14) | 12.728 ± 1.338 (14) |

No statistically significant differences observed in relation to fetal size or sex. Abbreviations: BV=Blood Vessel; GD=Gestational Day; CTMLW=Closest to Mean Litter Weight.
