# Supplementary figures and images for: Associations between fetal size, sex and placental angiogenesis in the pig
Source: Biol Reprod. 2018 Aug 18;100(1):239–52. doi: 10.1093/biolre/ioy184 (PMC6335214; doi:10.1093/biolre/ioy184)

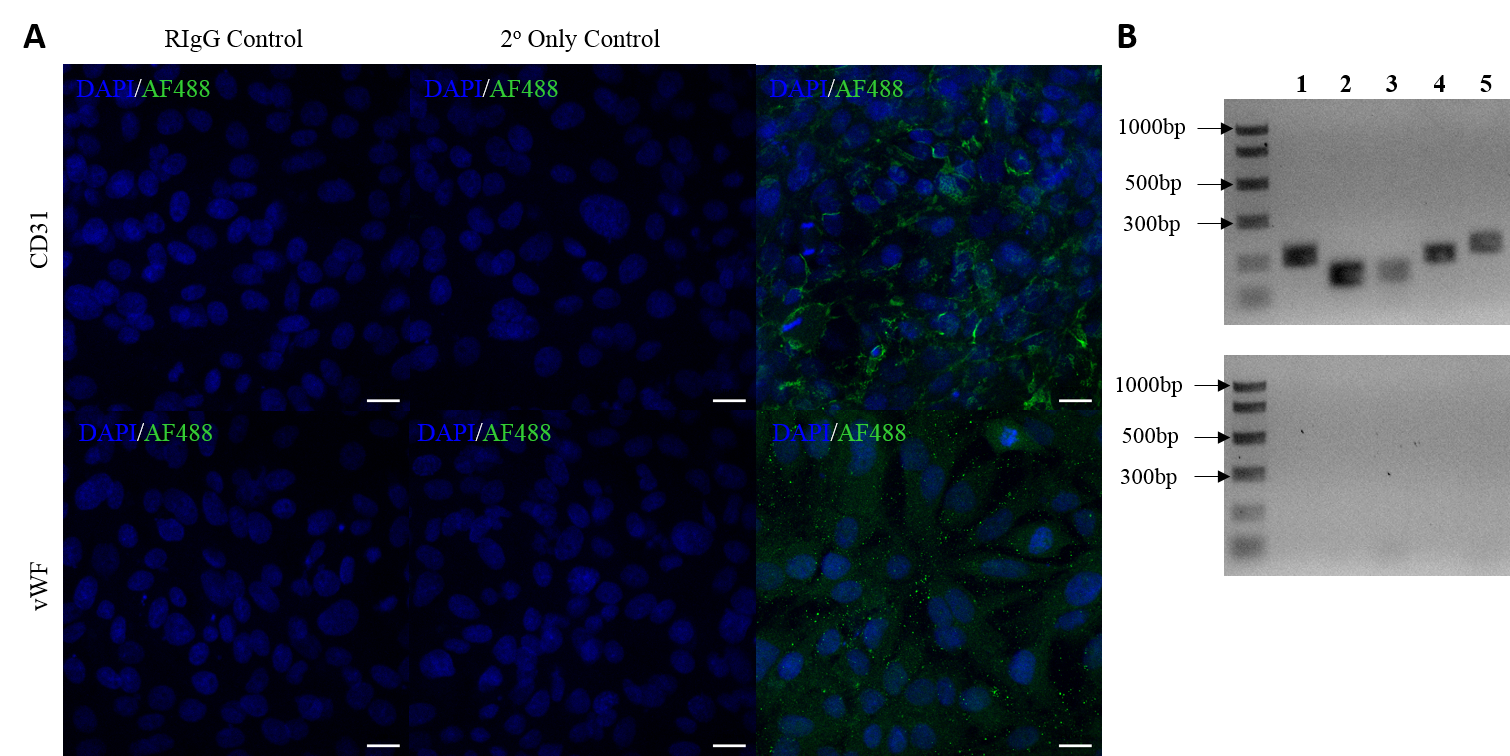

Supplement: Supplemental Tables and Figures [file ioy184_supplemental_tables_and_figures.zip › Supplementary Figure 3.tif]
